# Alexander Ogilvy

**Published:** 1914-06

**Authors:** 


### Obituary.

### ALEXANDER OGILVY, B.A., B.Ch., M.D., F.R.C.S.I.

Alexander Ogilvy was born in Dublin on November 18th, 1863, and his death on June 10th, 1914, at a comparatively early age, and in the height of popularity and success, is deeply deplored by all his colleagues and numerous friends.

For the following notes on his career we are indebted to his colleague, Mr. F. Richardson Cross :—

"After studying in the Coleraine Academical Institute, Dr. Ogilvy was entered at Trinity College, Dublin. He was a football player, but he was a better waterman, and most enthusiastic in any kind of yacht or sailing boat.

"He took the degrees B.A., B.Ch. and M.D., and was a Fellow of the Royal College of Surgeons of Ireland. He showed an early leaning towards ophthalmic surgery, and soon became House Surgeon to the National Eye and Ear Hospital in Dublin, where Fitzgerald, Sir Henry Swanzy and Storey were at work.

"In 1890 he succeeded Herman-Snellen as House Surgeon to the Bristol Eye Hospital, and remained at that post nearly three years, with, as the Report says, 'a most successful tenure of office.' He was then advised to go to Germany, and worked in the Vienna Clinic under Fuchs, and at Heidelberg under Leber. He also spent some time in London as Clinical Assistant in the Moorfields Ophthalmic Hospital. But he always had a strong attachment to Bristol, where he had already made many friends. He attended the meeting of the British Medical Association in Bristol in 1894, and expressed a desire to recommence work there. He arranged with Mr. Cross to return as his private assistant, and to help in the work of the Eye Department of the Royal Infirmary and the Eye Hospital. He studied steadily, and did a considerable amount of good pathological work with Dr. (now Colonel) Kelly, who was House Surgeon at the Eye Hospital at that time.

" In 1897 he started in practice for himself, but continued to act as Clinical Assistant in the two institutions mentioned. He then joined Mr. Prichard in the work of the Eye Dispensary in Orchard Street, and became one of the Surgeons there. His increasing experience and ability, associated with a natural geniality and charm of manner, soon brought him many clients, and when in 1900 the important post of Ophthalmic Surgeon to the Royal Infirmary became vacant Ogilvy was appointed to it, and from this time his professional success was assured. His assistance was increasingly asked for by the members of his profession, not only in Bristol, but in the neighbouring districts.

" His teaching was very popular among the students, and his special knowledge was of great value to his colleagues. No man set a higher value on the best traditions of the Royal Infirmary than he. He was true to his colleagues, kind and attentive to his patients, and generous to the poor. On Dr. Hailes resigning his post on the Eye Hospital Staff, Dr. Ogilvy was at once elected to succeed him, and thus he became once more associated with the institution which first brought him to Bristol.

" His patients and the profession both had the greatest confidence in his opinions, and in his power of diagnosis. He was a thoroughly good, all-round oculist. He was much interested in disease of the cornea and other superficial conditions. He was a very successful operator, and performed with great success such difficult manipulations as the ' simple ' extraction of cataract and trephining for glaucoma, in which disease he could show an exceptionally good list of successful cases.

" He wrote many reviews and several papers for this *Journal*, and he often made valuable communications at the local branch meetings, and also to the *Transactions of the Ophthalmological Society*, among them a case of ' Epithelial Carcinoma of the Conjunctiva,' ' Tuberculous Iridocyclitis in a man of 75,' ' Ivory exostosis of Orbit,' etc., etc.

" He had many and varied activities outside his profession, and did much useful work in Bristol. He was a popular and well-known figure, and with his kindly courtesy, cheerfulness, and keen sense of humour, he made friends wherever he went, and his society was much sought after. He was an energetic member of the well-known No. 6 Battery of the old Gloucestershire Artillery, and he did much both in the ranks and afterwards as an officer to help the work of the Corps to be pleasantly and effectually carried on. He was an excellent type of the genial, light-hearted Irish gentleman. He was a good actor, and the writer remembers an amusing incident when Dr. Ogilvy was playing 'Sir Lucius O'Trigger' in *The Rivals*, without any strain, quite naturally, and as part of himself, a critic said he had overdone the character by making it too Irish, both in speech and manner.

"As an instance of his popularity and ability he was elected a member of the Clifton Club Committee for many years, and three years at a time and for nine years in sequence he was chosen to be Chairman. He gave up a large amount of time to the interests of the Club; he was the means of several improvements in its management, and conducted its affairs with great tact and judgment He was a regular player on the Golf Links at Failand, and in May, 1913, he had the honour of being selected as Captain of the Club and Chairman of the Committee. A difficult bit of work had to be done, the raising of the annual subscription to the Club, and it was said to be entirely owing to his clever management that this was effected without opposition.

"About this time he had undergone a very serious operation for trouble that would have lowered the spirits of most men. It was faced with exceptional courage, and his self-control and hopefulness greatly helped to assist his cure. Much was expected from him, as his recovery was complete, but unhappily we have now instead to mourn the loss of a genial and reliable colleague, of a sound adviser, and of a bright and cheering companion, and to express our sincere sympathy with his wife in her sad bereavement, and with his many friends and patients."

The Rev. Canon Haigh (Vicar) conducted the funeral service, held in St. Paul's Church, Clifton, where there was a large attendance, the medical profession being particularly well represented.

Canon Haigh gave a brief and touching address, in which he said that in our hours of pain the noble and beautiful were never far away from us. At the post of duty there fell their dear brother, Dr. Alexander Ogilvy. Those who represented the profession of which he was so distinguished a member were there, owning all that he was and all that they were. English people were learning more and more to admire public men. They saw what beautiful gifts they had, and how lovingly they laid them at the feet of the public. They knew how they tended the poor. How many there were who could not be there who knew all they owed to Alexander Ogilvy. Now he had been taken from them, with his sweet manner and sympathy, his loving-heartedness and great-heartedness—a beautiful trait in anyone's character.

